# Training and supervision of lay mental health workers in community-based interventions in East Africa: a scoping review

**DOI:** 10.1080/16549716.2026.2688571

**Published:** 2026-06-24

**Authors:** Rosco Kasujja, Ronald Asiimwe, Barbra Makumbi, Cosmas Goodluck, Mariam Umugwaneza, Ibrahim Luberenga

**Affiliations:** a Department of Mental Health and Community Psychology, Makerere University, Kampala, Uganda; b Department of Family Social Science, University of Minnesota, Minneapolis, USA; c Department of Counselling and Higher Education, Ohio University, Athens, USA; d Department Human Development and Family Studies, Michigan State University, East Lansing, USA

**Keywords:** Task-sharing, mental health workforce, implementation science, capacity building, low- and middle-income countries

## Abstract

Lay mental health workers play a central role in implementing and sustaining mental health interventions across East Africa. However, limited research exists on how these workers are trained and supervised, an important gap given their role in addressing the region’s severe treatment shortage, where up to 85% of individuals with mental disorders receive little or no evidence-based care. Task-sharing, which involves training non-specialist workers to deliver psychological interventions under supervision, has become a key strategy over the past two decades. Yet, the processes that ensure LMHW competence, fidelity, and wellbeing, particularly training and supervision, remain underexplored. Guided by Arksey and O’Malley’s five-stage framework, this scoping review maps and synthesizes literature on training and supervision practices for LMHWs delivering community-based mental health interventions in East Africa. Systematic searches of five databases (Web of Science, Embase, Scopus, PubMed, and ProQuest) yielded records that, after screening and eligibility assessment, resulted in 18 included studies from five of the seven East African countries. Data were charted and thematically analyzed to describe training methods, supervision strategies, outcomes, and implementation challenges. Findings revealed wide variations in training duration and pedagogy, from brief workshops to multi-module or apprenticeship models, and diverse supervision formats, including hierarchical, peer, hybrid, and digital approaches. Evidence showed improvements in client outcomes, provider competence, and service delivery, but highlighted persistent gaps in standardized supervision, sustainability, and workforce support. This review informs policy and implementation efforts to strengthen LMHW training and supervision systems, contributing to narrowing the mental health treatment gap in East Africa.

## Paper Context

- **Main finding:** Training and supervision of lay mental health workers across East Africa vary widely in duration, content, and structure, yet remain central to improving community mental health service delivery.
- **Added knowledge:** This review maps, for the first time, how lay mental health workers in East Africa are trained and supervised, highlighting gaps in standardization, long-term support, and sustainability.
- **Global health impact for policy and action:** Strengthening and harmonizing training and supervision systems for lay mental health workers can enhance mental health workforce capacity, reduce treatment gaps, and inform global task-sharing policies in low-resource settings

## Background

Mental, emotional, neurological, and substance use disorders represent a growing share of the global disease burden, yet they remain among the most-neglected health concerns, particularly in low- and middle-income countries [1–3]. In sub-Saharan Africa, including East Africa, estimates suggest that up to 85% of individuals with mental disorders receive minimal or non-evidence-based care, reflecting a severe treatment gap [4]. East African countries, such as Uganda, Kenya, Rwanda, and Tanzania, face a critical shortage of mental health specialists. While psychiatrist-to-population ratios are often below 1 per 100,000 [5], there are also very few clinical psychologists, counseling psychologists, and other behavioral health professionals available to meet the population’s mental health needs, highlighting the importance of including diverse mental health voices in care delivery. This acute human resource deficit has led to global and regional policy calls for task-sharing approaches, in which non-specialist and lay health workers have been utilized to deliver basic mental health interventions [e.g. psychoeducation, problem-solving therapy, and behavioral activation] under appropriate supervision [6,7].

Over the past two decades, lay mental health workers [LMHWs] have played a critical role globally in extending care to underserved communities, including in low- and middle-income countries such as those in East Africa [8]. Importantly, LMHWs are also increasingly embedded within community-based mental health rehabilitation services, where they support psychosocial rehabilitation, functional recovery, and reintegration of individuals with mental health conditions into family and community life. In East Africa, lay mental health workers with minimal formal training but strong community ties have been trained to deliver evidence-based psychological interventions, including IPT, CBT, PST, and GSP [9]. Adapted group versions of IPT have been successfully implemented in Uganda to support individuals experiencing depression and grief [10]. Landmark examples of lay-delivered mental health interventions in sub-Saharan Africa include the Friendship Bench in Zimbabwe [11], the Group Support Psychotherapy model in Uganda [12], and the community-based social healing model in Rwanda, implemented by the Ubuntu Center for Peace, which trains lay leaders, referred to as Community Healing Assistants to facilitate therapeutic groups following a 15-week training program [13]. These programs have demonstrated significant improvements in depression, psychosocial functioning, and adherence when delivered by trained lay providers.

While the promise of LMHWs is widely acknowledged, questions remain about training and supervision, which are central to ensuring intervention fidelity, quality of care, and sustainability [7]. Implementation science evidence indicates that brief, structured training combined with ongoing supportive supervision can significantly improve LMHW knowledge, confidence, and effectiveness [14,15]. However, supervision models are inconsistently reported, under-evaluated, and often not adapted to fragile health systems in East Africa. Beyond training and supervision, the effectiveness of LMHW-led interventions is also shaped by broader sociopolitical and structural factors in East Africa, including poverty, conflict and displacement, stigma surrounding mental illness, and uneven investment in mental health systems. These contextual realities influence both service delivery and help-seeking behaviors and can either constrain or enable the success of community-based mental health programs. A lack of or inadequate supervision risks provider burnout, attrition, and reduced quality of care. Moreover, cultural, ethical, and logistical factors such as literacy levels, community norms, and health system integration influence the success of LMHW training programs.

Existing systematic and scoping reviews highlight the global evidence for task-sharing, yet there has been limited synthesis focusing specifically on East Africa, where the mental health burden is compounded by poverty, stigma, conflict, and health system fragility [16,17]. This gap underscores the need for a regional synthesis that examines how LMHWs are trained and supervised, what models are most effective, and what barriers and facilitators exist in community-based mental health interventions. Although task-sharing models have been widely studied across low- and middle-income countries, important contextual differences exist across regions in terms of health system organization, workforce capacity, and sociocultural factors. East Africa presents a particularly important context for focused synthesis due to its high burden of mental health needs, severe workforce shortages, and the rapid expansion of community-based mental health initiatives. A regional focus allows for more contextually relevant insights that may be obscured in broader LMIC-level analyses.

Given the gap in science highlighted above, this scoping review seeks to map and synthesize the available literature on the training and supervision of lay mental health workers in community-based interventions across East Africa. The review focuses specifically on post-training implementation processes, particularly supervision, follow-up support, and applied practice, while acknowledging that in many contexts training is an ongoing and iterative process rather than a one-time event. The review is guided by three research questions: (1) What training approaches are used to prepare/train LMHWs for delivering mental health interventions in East Africa? (2) What supervision strategies are implemented to support LMHWs in these contexts, post training? and (3) What are the reported outcomes, challenges, and facilitators of training and supervision models

By consolidating this evidence, the review will inform strategies to strengthen the quality of training and supervision of LMHWs, as well as the sustainability and scalability of LMHW-delivered interventions in East Africa, ultimately contributing to closing the mental health treatment gap in the region.

## Methods

This scoping review followed the methodological framework proposed by Arksey and O’Malley [18], which outlines five stages: identifying the research questions, identifying relevant studies, study selection, charting the data, and collating, summarizing, and reporting the results. The framework was applied with minor methodological refinements informed by subsequent scoping review guidance, including enhanced reporting standards and use of systematic review management software to support study selection and data charting.

### Identifying the research questions

The research idea was conceptualized by the first and last authors. The two authors met to develop and refine the review focus. An initial review of the literature on the implementation of mental health interventions by lay mental health workers (LMHWs) in African settings was conducted to inform the scope of the review. The final review questions were derived through iterative discussion among the research team based on identified gaps in the literature and the overall aim of the study. These include;

- What training methods are used to prepare LMHWs for delivering mental health interventions in East Africa?
- What supervision strategies are in place to support LMHWs in these settings?
- What are the reported outcomes, challenges, and facilitators of training and supervision models?

### Identifying relevant studies

A search strategy for this study was developed by the last author, following the Population Concept Context [PCC] framework [19] to search for articles across different databases. Boolean operators were also used during the article search process to ensure that no relevant studies were overlooked. The initial search was conducted on the Web of Science, using the following terms: ‘Lay health worker’ OR ‘community health worker’ OR ‘peer provider’ OR ‘paraprofessional’ AND [‘mental health’ OR ‘psychosocial support’] AND [‘training’ OR ‘capacity building’ OR ‘education’] AND [‘supervision’ OR ‘mentoring’ OR ‘support’] AND [‘community-based’ OR ‘task-shifting’] AND [‘East Africa’ OR Uganda OR Kenya OR Tanzania OR Rwanda OR Ethiopia OR ‘Sub-Saharan Africa’]. Then, the search strategy and terms were adapted for other databases based on each database’s guidelines to avoid missing relevant articles. The databases searched included Web of Science, Embase, Scopus, ProQuest, and PubMed, selected for their coverage of peer-reviewed literature on mental health interventions. The search was conducted on 15 June 2025. Gray literature (e.g. reports, theses, and non-indexed program documents) was not systematically searched; however, this decision was made to ensure consistency in methodological quality and comparability across included studies.

### Study selection

After conducting the search, all identified articles were uploaded to Covidence (Veritas Health Innovation, Melbourne, Australia; available at www.covidence.org), where duplicate removal and screening were conducted. The system automatically detected and removed 15 articles because they were duplicates. Two independent reviewers conducted title and abstract screening using predefined inclusion and exclusion criteria. To standardize the process, a pilot screening exercise was undertaken on a subset of studies to ensure consistency in the interpretation of the criteria. Inter-rater agreement was assessed using the percent agreement. Discrepancies were resolved through discussion between reviewers, and where consensus was not reached, the first and last authors adjudicated the final decision based on the established eligibility criteria.

### Inclusion and exclusion criteria

During this stage of the review, specific inclusion and exclusion criteria were carefully applied to guide the selection of studies. Eligible studies were those conducted in East African countries, including Uganda, Kenya, Tanzania, Rwanda, Burundi, Ethiopia, South Sudan, and Somalia. The interventions examined needed to be community based and involve lay or local mental health workers [LMHWs], with a clear focus on training and/or supervision practices. Additionally, the review was limited to studies published in English, which may have excluded relevant research and programmatic reports available in local languages. This is particularly important in the East African context, where a substantial proportion of mental health interventions are implemented and documented by non-governmental organizations and community-based programs, often outside peer-reviewed literature. As a result, some context-specific insights and implementation experiences may not have been captured in this review. Studies were excluded if they focused solely on facility-based professional mental health workers, addressed interventions unrelated to mental health or not rooted in community settings, or failed to discuss elements of training or supervision.

**Figure 1.**
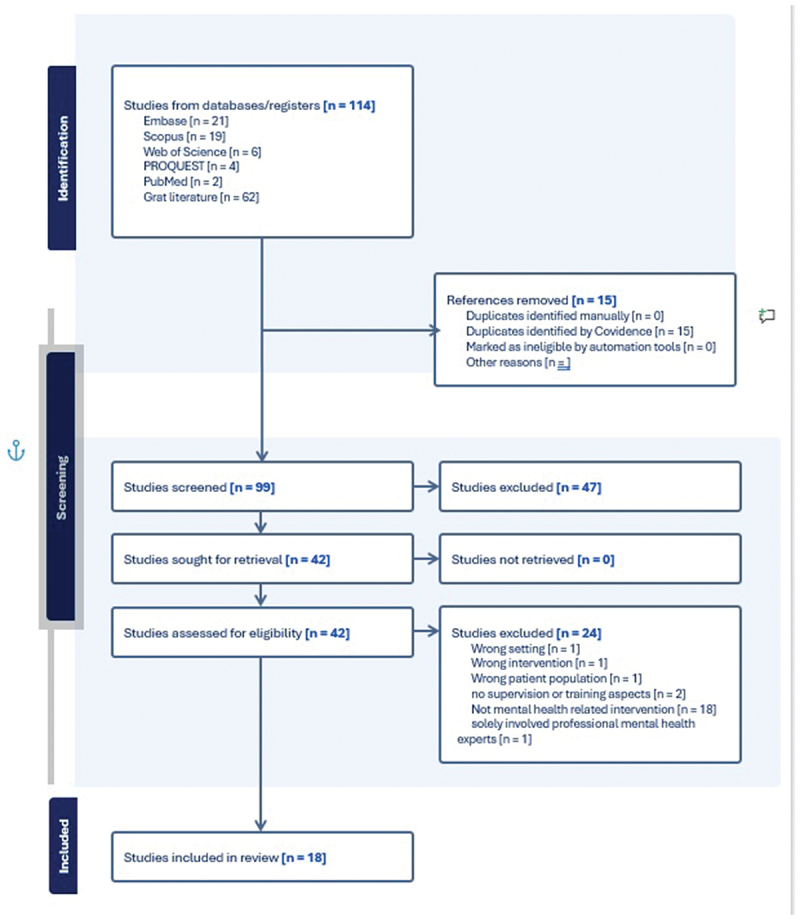
PRISMA diagram mapping the process of article screening.

After the abstract and title screening, 42 articles passed the initial review and were then retrieved for full-text examination. Three members of the research team, the second, third, and fourth authors, led the full-text review process. The other two authors, the first and second, met to discuss and resolve any discrepancies when the two reviewers had different votes on a given article. This process concluded when 24 articles were excluded because they did not meet the inclusion criteria, leaving 18 articles for data extraction (see Figure 1).

### Charting the data

Based on the research questions, an extraction template was created by the last author (IL) and shared with the team for consensus, who provided feedback on whether it captured the key information from the articles. A finalized version was then distributed to initiate the extraction process. All team members participated in extracting data, but an independent reviewer who did not extract an article examined the work of others to compare notes and reach a consensus on the final data. This approach was designed to promote transparency and ensure that no crucial information was overlooked during this critical stage.

### Collating, summarizing, and reporting the results

Research questions one and two were presented in tables accompanied by a narrative explanation of the results. The third question was addressed using tables along with content analysis, which facilitated the identification of themes.

## Results

This scoping review aimed to map and synthesize evidence on the training and supervision of lay mental health workers delivering community-based mental health interventions in East Africa. The findings presented below summarize the key training approaches, supervision strategies, outcomes, and implementation challenges identified across the included studies.

### Approaches to training lay mental health workers in East Africa

Across the reviewed studies, various training methods were used to prepare lay and non-specialist mental health workers [LMHWs] in East Africa (Table 1). Training content ranged from basic orientations to comprehensive multi-module workshops, often customized to the specific intervention needs [20]. In East Africa, mental health services are often delivered within broader health systems, particularly HIV care programs, where longstanding investments in community-based service delivery have created platforms for integrating mental health support.

**Table 1.** Training approaches are used to prepare LMHWs for delivering mental health interventions.

Study	LMHW Type	Training Content	Duration/Frequency	Methods Used	Facilitators
Knettel [20]	Community Health Workers (CHWs)	Preliminary training on the current landscape of HIV care given recent advancements, developing underemphasized skills such as counseling skills, and stigma reduction.	Only preliminary training when they began their roles.	Not specified in the provided excerpts.	Not specified in the provided excerpts.
Lifson [21]	Trained peer Community Health Support Workers (CHSWs) who were HIV positive and from the same neighborhood/village as the patient.	HIV and health education, counseling/social support, and facilitation of communication with HIV clinics.	Initial training was provided to all CHSWs before they began their work. Refresher training sessions were conducted periodically during the 12-month program.	Didactic lectures on HIV, treatment, nutrition, and health-promoting behaviors. Role-playing and group exercises focused on counseling skills and providing empathetic support.	Supported by the Ethiopian team of NASTAD and collaborating public health professionals.
Nakimuli-Mpungu [12,22]	Trained lay health worker.	For Group Support Psychotherapy (GSP): Intensive 5-day workshop covering eight modules such as an overview of the GSP model, depression and HIV/AIDS, basic counseling and coping skills, livelihood skills (enterprise selection, financial literacy, resource mobilization), and self-care strategies. For Group HIV Education (GHE): Focused on depression and HIV/AIDS, HIV progression, transmission, mother-to-child transmission, and basic antiretroviral therapy facts.	The training for both interventions was conducted over a 4-month period using a training-of-trainers model. GSP formal training lasted 5 days. GHE training lasted 2 days. Both were followed by informal training through supervised pilot sessions conducted between May and August 2016. Additionally, for GSP, once a week for 8 weeks.	For GSP: Active learning methods like role plays, group discussions, and brainstorming. For GHE: Delivered in lecture format. Both followed by supervised pilot sessions. A training-of-trainers model was used.	Mental health specialists and HIV care providers. For GSP, mental health specialists from Makerere University in collaboration with the Ministry of Health trained primary health care (PHC) workers, who then trained the LHWs. For GHE, HIV care providers from The AIDS Support Organization (TASO) served as the trainers.
Brathwaite [23] & SensoyBahar [24]	Parent peers (MFG-PP) and community health workers (MFG-CHW). Lay CHW.	Intervention content (4Rs and 2Rs), and facilitation skills. Six main components: the 4Rs (Rules, Responsibilities, Relationships, Respectful communication) and 2Ss (Stress and Social Support).	The intervention comprised 16 sessions. Facilitators were required to score 85% or above on the Knowledge Skills and Attitude Test (KSAT) after training. Not specified.	A combination of didactic instruction, role-playing, and practice sessions.	Team members trained by the study’s Principal Investigator (PI) who developed the original intervention. Trained trainers who observed all sessions and provided feedback to facilitators.
Klein [25]	Lay leaders.	Role-playing group sessions. Review of how to approach potential issues during sessions (e.g. distress upon revisiting a trauma memory). Focused on teaching leaders how to effectively lead discussions.	2-day training that lasted a total of approximately 8 hours.	Role-playing group sessions.	The supervision team, which consisted of clinical psychologists and doctoral students affiliated with academic institutions.
Ibrahim [31]	Community health workers, particularly female health workers (Marwo Cafimad).	Training is proposed as part of the recommended intervention. Emphasizes the need for training, but does not include implementation details.	Emphasizes the need for training, but does not include implementation details.	This information is not provided in the source.	This information is not provided in the source.
Tilahun [29]	Community Health Extension Workers (HEWs).	Child Mental Health, Common Child Mental Disorders, Case Identification and Referral, Community Awareness and Stigma Reduction, Communication and Engagement Skills, Basic Intervention Strategies.	Total mental health training: 2 weeks (10 sessions). Child mental health content: 1 day (1 session). Frequency: One-time training during the diploma upgrading program.	Face-to-face lectures supported by printed training modules.	Likely to be health educators or trainers assigned by the Federal Ministry of Health (FMOH) of Ethiopia in collaboration with the Open University UK, regional health bureaus, or partnering academic institutions.
Murray [32]	Lay counselors.	Foundational mental health knowledge, active and experiential learning of evidence-based counseling strategies, and supervisor-specific skills to ensure sustainability.	Not specified.	Apprenticeship model. Experiential, skills-based learning and coaching. Multi-modal, combining in-person, virtual, observational, and structured documentation-based strategies.	Developed and used by the authors themselves. For the Ugandan case study, supervisors included local psychiatrists or staff already involved in psychosocial programs.
Bond [30]	Nonspecialist community providers and Community-Based Volunteers (CBVs) like peers, community health workers, or volunteers.	Topics related to Sugira Muryango; child development and how families should relate to each other, advantages of early stimulation and how to handle different issues without causing trouble.	3-week training was conducted prior to intervention delivery. Refresher training was frequently mentioned as helpful for reinforcing knowledge over several months of delivery.	Not explicitly detailed in the provided excerpts.	Most supervisors were affiliates of the local implementing partner, FXB Rwanda, and had helped develop training and monitoring tools.
Barnett [31]	Non-specialist providers like peer volunteers and health workers.	Tailoring training to the skills and needs of the non-specialist provider and their roles.	The article discusses ‘how initial and ongoing training are conducted’, but specific durations or frequencies are not provided in this excerpt.	Not specified in this excerpt.	Not specified in this excerpt.
Venturo-Conerly [28]	Lay providers.	General Skills for Group Leadership. Teaching lay providers how to handle group dynamics, address student questions, and stay on protocol to ensure smooth session facilitation. Peer Counseling Skills.	Training lasted for 10 hours over 2 days.	Not explicitly detailed beyond stating skills.	Study Team Members, including a core team from Nairobi (T.O., D.N., C.M., V.M., and C.W.). Doctoral-level researchers and clinicians (such as D.N., C.M., V.M., C.W., and A.B.).
Wahida 2019	Primary health care providers (PHCPs) – a clinical officer, nurses, and a midwife.	Assessment, management, and treatment of common mental disorders as summarized from the Uganda Clinical Guidelines (UCG).	Training and support supervision were provided over a 12-week period as part of the 6-month (24 weeks) intervention. The intervention included 12 weeks of active training and support supervision by a mental health specialist.	Didactic training sessions (teaching the summarized Uganda Clinical Guidelines (UCG) on common mental disorders). Practical coaching and real-time support supervision.	Mental health specialists from Mbarara Regional Referral Hospital.
Karungi [34]	Lay Health Workers (LHWs).	Four core competency domains in the WHO dementia toolkit. Analysis of the intervention concentrated on needs assessment, early detection and management, community engagement, and support for people with dementia.	The training itself was for 5 days. This was followed by eight weeks of implementation of the learned skills.	Not explicitly detailed beyond the focus areas.	Not specified in the provided excerpts.
Asher [36]	Lay health workers.	Not explicitly detailed in the yellow highlights.	Not explicitly detailed in the yellow highlights.	Not explicitly detailed in the yellow highlights.	Not explicitly detailed in the yellow highlights.
Mutamba 2013	Lay community health workers (LHWs).	This information is not provided in the source.	This information is not provided in the source.	This information is not provided in the source.	This information is not provided in the source.
Wall [26]	CHW (Lay counselors).	Delivering the Tuko Pamoja (TP) family therapy intervention.	60 hours of training focused on delivering the Tuko Pamoja (TP) family therapy intervention.	Not mentioned, most likely session feedback.	Local supervisors received weekly consultation from clinical psychologists to support their supervisory role.

Lay mental health workers are frequently embedded within these systems, providing psychosocial support, adherence counseling, and stigma reduction alongside clinical services [20,21]. This interrelationship reflects the bidirectional links between mental health and HIV outcomes, where mental health challenges can affect treatment adherence and disease progression, while living with HIV can increase vulnerability to psychological distress. As a result, training and supervision of LMHWs in this context often span both mental health and HIV-related competencies. More structured training programs were observed in Nakimuli-Mpungu [12,22], where lay health workers participated in an intensive five-day Group Support Psychotherapy [GSP] workshop covering eight modules, along with 2 days of Group HIV Education [GHE].

These lectures, combined with role plays, group discussions, and supervised pilot sessions, were delivered through a training-of-trainers model led by mental health specialists and HIV care providers. Similarly, Brathwaite [23] and Sensoy-Bahar [24] trained parent peers and community health workers in the ‘4Rs and 2Ss’ model [Rules, Responsibilities, Relationships, Respectful communication, Stress, and Social support], requiring facilitators to attain competency scores of at least 85% after didactic instruction, role-plays, and practice sessions supervised by the intervention’s original developers.

Shorter, skills-based trainings were also documented in two studies [25,26]. For example, Klein [27] trained lay leaders in role-playing strategies and group facilitation over 2 days [8 hours], guided by clinical psychologists and doctoral students. Venturo-Conerly [28] also emphasized leadership and peer counseling skills through a 10-hour, two-day training delivered by researchers and clinicians. In Wall [26], CHWs received 60 hours of training to deliver the ‘Tuko Pamoja’ family therapy intervention, followed by ongoing supervision from clinical psychologists.

In other studies, interventions focused on child and family well-being. Tilahun [29] trained health extension workers in child mental health, common child disorders, referral pathways, stigma reduction, and basic interventions through a two-week diploma program with lectures and printed modules, delivered by trainers from Ethiopia’s Federal Ministry of Health and academic partners. Bond [30] similarly trained nonspecialist community providers and volunteers in family functioning and early child stimulation through 3 weeks of initial training with refresher sessions, supported by supervisors from FXB Rwanda. Barnett [31] also emphasized tailoring training content to the skills of non-specialist providers, although specific durations were not detailed.

Three studies focused on broader system-level skills. Murray [32] implemented an apprenticeship model that combined experiential learning, coaching, observation, and documentation to prepare lay counselors and supervisors. Wakida [33] trained primary healthcare providers on common mental disorders over 12 weeks, using a combination of didactic sessions, practical coaching, and ongoing supervision from mental health specialists. Karungi [34] trained lay health workers over 5 days on dementia care competencies, followed by 8 weeks of supervised practice. A few studies provided limited detail, emphasizing the need for training without offering implementation specifics or reporting how learning outcomes or competencies were assessed, making it difficult to evaluate the effectiveness of the training models [35,36].

### Supervision strategies used to support LMHWs

The studies reviewed highlighted both diversity and inconsistency in how supervision for lay health workers [LHWs] and community-based providers was organized and carried out across East Africa (see Table 2). Supervision models varied from hierarchical approaches, where professional clinicians or trained supervisors oversee LHWs [26,27,29], to peer-based or team-focused approaches that prioritize observation, group feedback, and collaborative learning [23,33]. A few studies, such as Sensoy-Bahar [24], show hybrid approaches that mix hierarchical oversight with daily peer and team support, indicating a trend toward mixed models that combine accountability with collaborative learning.

**Table 2.** Supervision strategies are implemented to support LMHWs.

Study ID	Study Title	Supervision Type	Frequency	Mode	Supervisor Role	Tools Used
Knettel [20]	Patient perspectives on the helpfulness of a community health worker program for HIV care engagement in Tanzania	Not specified	Not mentioned	Not mentioned	Not mentioned	Not mentioned
Lifson [21]	Implementation of a Peer HIV Community Support Worker Program in Rural Ethiopia to Promote Retention in Care	not specified in the provided excerpts.	not specified in the provided excerpts.	not specified in the provided excerpts.	Supervision was supported by the Ethiopian team of NASTAD and collaborating public health professionals.	Not mentioned
Nakimuli-Mpungu [22]	The effect of group support psychotherapy delivered by trained lay health workers for depression treatment among people with HIV in Uganda: Protocol of a pragmatic, cluster randomized trial	Supervisors conducted direct observation and assessed LHW counseling skills during 3 to 4 group sessions using standardized checklists adapted from the Enhancing Assessment of Common Therapeutic Factors scale. LHWs also completed self-administered feedback questionnaires after each session, which supervisors reviewed to identify challenges and provide guidance.	Supervision was provided during 3 to 4 group sessions for each lay health worker as they delivered the intervention, with supervisors regularly reviewing session feedback to monitor progress and address any challenges.	In-person, with trained primary health care workers directly observing and assessing lay health workers during group sessions. Additionally, supervisors reviewed written feedback questionnaires completed by the lay health workers after each session to guide ongoing support.	The supervisors were trained primary health care (PHC) workers who had received prior training from mental health specialists or HIV care providers.	A supervision checklist adapted from the Enhancing Assessment of Common Therapeutic Factors scale to assess the lay health workers basic counseling skills. Additionally, supervisors reviewed self-administered semistructured feedback questionnaires completed by the lay health workers after each group session, which detailed session content and noted facilitators and barriers encountered.
Brathwaite [23]	Short-Term Impact of Amaka Amasanyufu Multiple Family Group Intervention on Mental Health Functioning of Children With Disruptive Behavior Disorders in Uganda	During intervention delivery, trainers observed all sessions and provided feedback to facilitators	all sessions were observed	Implied in-person observation and feedback	trainers/team members	Knowledge Skills and Attitude Test (KSAT)
Klein 2023 [25]	WhatsApp supervision for a layâled Islamic trauma-focused intervention in Somaliland: Qualitative content analysis	Hierarchical, with clinical psychologists supervising lay leaders, conducted in a group format. A supportive approach was used too	Weekly	Digital/Remote WhatsApp text	Clinical psychologists (three licensed) and doctoral students in clinical psychology	WhatsApp text messages, pre planned messages
Ibrahim [31]	Investing in mental health in Somalia: harnessing community mental health services through task shifting	Not specified in the provided excerpts	Not specified in the provided excerpts	Not specified in the provided excerpts	Not specified in the provided excerpts	Not specified in the provided excerpts
Tilahun [29]	Training needs and perspectives of community health workers in relation to integrating child mental health care into primary health care in a rural setting in sub-Saharan Africa: a mixed methods study		No supervision frequency reported or documented.	Blended approach of direct, in-person lectures combined with printed educational resources.	Most likely trained instructors or health educators affiliated with the training centers (e.g. Colleges of Health Sciences at Hawassa and Hossana).	Not documented
Nakimuli-Mpungu [12]	Effectiveness and Cost-Effectiveness of Group Support Psychotherapy Delivered by Trained Lay Health Workers for Depression Treatment Among People with HIV in Uganda: A Cluster-Randomized Trial	The description indicates that supervision was part of a structured process involving structured health worker training, ongoing supervision, and support activities aimed at ensuring treatment fidelity. These activities were likely managed in a hierarchical or team-based framework, where trained supervisors provided support to LHWs and healthcare workers to maintain intervention quality and fidelity.	Group sessions were led by trained lay health workers once a week for 8 weeks, but the article does not specify how often supervision was provided to the health workers and LHWs. It mentions that supervision was part of the ongoing support and supervision activities to ensure treatment fidelity, but the text does not provide details about how often these supervision sessions occurred.	Possibly in-person training and support sessions, complemented by ongoing monitoring and oversight to maintain quality and fidelity of intervention delivery.	The health workers who facilitated the training of trainers.	Structured training manuals and guidelines.
Murray [32]	Building capacity in mental health interventions in low resource countries: an apprenticeship model for training local providers.	Apprenticeship Model is experiential, skills-based learning and coaching	Not specified	Multi-modal, combining in-person, virtual, observational, and structured documentation-based strategies to support skill development, fidelity, and long-term sustainability in LMIC settings.	For the Ugandan case study supervisors included local psychiatrists or staff already involved in psychosocial programs.	Not specified
Bond [30]	Exploring Nonspecialist Preparedness to Deliver an Evidence Based, Family Strengthening Intervention in Rwanda: A Qualitative Study	The supervision primarily involved a hierarchical model where CBVs were assigned a designated supervisor. There was also an element of peer learning through weekly group sessions	Regular supervision was intended to support CBVs on a weekly basis Supervisors held monthly group supervision sessions with all CBVs in a geographic cell	in-person, phone	Supervisors had a bachelors degree in clinical psychology or social work	Sugira Muryango manual, tablets, Audio recorders, Quality of delivery checklist
Barnett [31]	Effective training practices for non-specialist providers to promote high-quality mental health intervention delivery: A narrative review with four case studies from Kenya, Ethiopia, and the United States	Not specified in this excerpt.	Not specified in this excerpt.	Not specified in this excerpt.	Not specified in this excerpt.	Not specified in this excerpt.
Venturo-Conerly [28]	Effective training practices for non-specialist providers to promote high-quality mental health intervention delivery: A narrative review with four case studies from Kenya, Ethiopia, and the United States	Not specified in this excerpt.	Not specified in this excerpt.	Not specified in this excerpt.	Not specified in this excerpt.	Not specified in this excerpt.
Sensoy-Bahar [24]	Training and Supervising Lay Providers in Kenya: Strategies and Mixed-Methods Outcomes	The supervision integrated hierarchical oversight with team-based collaboration.	Weekly Supervision Meetings: These involved all available members of the study team and all lay providers, occurring twice a week for 30 minutes, where they reviewed upcoming sessions and addressed concerns. Daily supervision: Each lay provider was assigned a daily supervising member of the study team at each school. This member of the study team provided their assigned lay providers with materials, collected materials from the lay providers after the sessions, gave the lay providers time warnings at least once per session, and were available for the lay providers to call over if they had any questions or concerns during the groups.	The supervision mode combined in-person meetings and oversight with ongoing digital communication via WhatsApp, constituting a hybrid mode of supervision.	The supervision was led by a study team supervisor for the day, who coordinated session reviews and addressed concerns at each school during intervention days. Doctoral-level researchers and clinicians (such as D.N., C.M., V.M., C.W., and J.W.), who provided oversight and support to the undergraduate team members organizing training and supervision activities. Undergraduate team members (including K.V.-C., E.R., and T.O.) delivered the training and conducted supervision under regular support and guidance from these doctoral-level advisors.	Structured review meetings: These included weekly supervision meetings of 30 minutes each, occurring twice a week) where the entire study team and all lay providers discussed session content, addressed concerns, and practiced upcoming protocols in small groups. Rubrics and role plays: To assess and improve performance, the study used feedback rubrics during role-plays, allowing trainers to evaluate providers’ adherence to protocols and identify areas for improvement. Providers received verbal feedback based on these rubrics. Follow-up practice sessions: For providers struggling with certain skills, the trainers arranged individual follow-up role plays (e.g. a 30-minute phone call) to practice specific skills and ensure readiness. Communication through WhatsApp: The study team used WhatsApp messages to send important reminders, updates, and support to lay providers during intervention delivery.
Wakida [33]	“I decided to participateâ€¦.because I saw it as benefiting our community and families: a qualitative study of lay providers experiences with delivering an evidence-based mental health intervention for families in Uganda	It was team-based, with SMART Africa trainers observing all sessions and providing feedback to the facilitators upon completion of each session.		in-person, as the SMART Africa trainers observed all sessions and provided real-time feedback afterwards.	trained trainers who observed all sessions and provided feedback to facilitators.	supervision tools included fidelity checklists and independent fidelity observations.
Karungi [34]	Implementing clinical guidelines to promote integration of mental health services in primary health care: a qualitative study of a systems policy intervention in Uganda	Supervision was provided by mental health specialists	Regular during the 12-week active intervention period.	In-person and on-site	Mental health specialists from Mbarara Regional Referral Hospital.	Summarized Uganda Clinical Guidelines (UCG) on common mental disorders â€” packaged as easy-to-use table charts to guide assessment and management. Modified Health Management Information System (HMIS) registers (031 for outpatient and 071 for antenatal) â€” updated to include specific columns for mental health disorders (depression, bipolar disorder, epilepsy, alcohol use disorders) to improve recording and monitoring. A clinician checklist, a wall chart pinned in consultation rooms, outlines step-by-step processes that clinicians should follow during patient assessments, including mental health evaluations. Supervisors use audit tools to review the modified registers and clinical documentation for accuracy and adherence to guidelines.
Asher [36]	Lay Health Workers in Community-Based Care and Management of Dementia: A Qualitative â€ and Intervention Study in Southwestern Uganda					
Mutamba 2013	Like a doctor, like a brother: Achieving competence amongst lay health workers delivering community-based rehabilitation for people with schizophrenia in Ethiopia	This information is not provided in the source.	This information is not provided in the source.	This information is not provided in the source.	This information is not provided in the source.	This information is not provided in the source.
Wall [26]	“What about lay counselors’ experiences of task-shifting mental health interventions? Example from a family-based intervention in Kenya	hierarchical, team-based	after each counseling session	In-person	local supervisors received weekly consultation from clinical psychologists to support their supervisory role	Not mentioned, most likely session feedback

The frequency of supervision varied widely, with some studies involving intensive and ongoing contact, including daily oversight [e.g. Sensoy-Bahar et al. [24], or weekly group check-ins [25,30], while others offer episodic supervision linked to intervention delivery [22,23]. This variation shows the lack of standardization in supervision methods and highlights the difficulty of balancing feasibility with fidelity in low-resource settings.

Regarding supervision methods, both in-person observation [22,33,34] and digital/remote strategies like WhatsApp supervision [24,25] were used, with digital approaches showing promise for sustainability in resource-limited and geographically dispersed settings. The tools used also vary widely, from structured checklists and fidelity rubrics [22,33,34] to digital manuals, role-play feedback, and mobile platforms [24,30].

Despite these innovations, significant gaps in the literature were identified. Few studies have thoroughly assessed the effectiveness of supervision in maintaining intervention quality and outcomes beyond descriptive reporting. There was limited attention to community involvement in the design and delivery of supervision, despite the community-based nature of many interventions. Similarly, long-term supervision strategies aimed at retaining lay health workers and preventing burnout were rarely examined, leaving important questions about sustainability unresolved. Overall, the evidence showed significant variation in supervision practices, with several studies reporting hybrid and technology-supported models.

### Facilitating factors in training and supervision models

Effective training and supervision models are fundamental to the successful implementation of community-based mental health interventions delivered by lay providers in low resource settings (see Table 3). Across studies, the training and supervision of lay mental health workers [LMHWs] in East Africa were facilitated by a range of actors, including academic institutions, health professionals, research teams, and government health systems.

**Table 3.** Outcomes, challenges, and facilitators of training and supervision models.

Study ID	Facilitators	Outcomes Reported	Outcome Measures	Impact on LMHWs	Impact on Community	Reported Challenges
Knettel [20]	Not specified	User Satisfaction, Retention in Care, and Improved Service Delivery	The study primarily used qualitative interview data to assess patient perceptions	engagement, stigma reduction, improved emotional well-being	Improved uptake of HIV services and quality of life among PLWH. â€¢ Enhanced quality and reach of HIV services through improved testing, care initiation, and retention. CHWs offer emotional support to accept one’s diagnosis and cope with stigma.	Limited Program Reach, Lack of CHW Engagement and Depth of Interactions, Socioeconomic Challenges, Insufficient CHW Training
Nakimuli-Mpungu [22]	The facilitators of the training were mental health specialists and HIV care providers. For the GSP training, mental health specialists from Makerere University, in collaboration with the Ministry of Health, trained primary health care (PHC) workers, who then taught the lay health workers (LHWs). For the Group HIV Education (GHE) training, HIV care providers from The AIDS Support Organization (TASO) served as the trainers using the same training-of-trainers approach.	The trial reported that GSP delivered by lay health workers led to significant reductions in major depression rates and improvements in functional status among persons living with HIV at 6 months post-treatment. Secondary outcomes showed positive effects on self-esteem, posttraumatic stress symptoms, social support, stigma, adherence to antiretroviral therapy, viral load suppression, disability days, and asset possession.	Outcome measures were collected through interviewer-administered standardized questionnaires at multiple time points: baseline, post-treatment, and at 6-, 12-, 18-, and 24-month follow-up.	The trial positively impacted LHWs by equipping them with knowledge and skills through training and supervision, enabling them to effectively deliver culturally sensitive group support psychotherapy to persons living with HIV and depression. This empowerment enhanced their competencies in counseling, facilitating social connections, and supporting coping strategies within their communities. The process evaluation highlighted how LHWs contributed to improving mental health outcomes and livelihood opportunities for participants, demonstrating their vital role in bridging mental health service gaps in low-resource primary care settings.	The trial intervention positively impacted the community by improving mental health and functioning among persons living with HIV, which in turn enhanced their ability to work, save, and accumulate livelihood assets. By addressing depression through culturally sensitive group psychotherapy delivered by lay health workers, the program helped break the cycle of poverty and poor mental health common in this vulnerable population. This community-level empowerment fostered greater social support, resilience, and independence, contributing to overall well-being and strengthening the integration of mental health services within existing HIV care platforms in resource-limited, rural settings.	Limitations in the generalizability of findings to other ethnic populations within Uganda and the difficulties in accurately measuring adherence to antiretroviral therapy (ART), as there is no established gold standard for adherence measurement. Delivering psychological interventions through LHWs in low-resource, rural settings posed challenges related to maintaining treatment fidelity, addressing cultural nuances, and managing logistical issues such as supervision and participant engagement.
Brathwaite [23]	Team members trained by the study PI.	Children in both intervention groups (MFG-PP and MFG-CHW) had significantly lower depressive symptoms and higher self-concept compared to children in the control group. Caregivers in both intervention groups had significantly lower caregiving-related stress and fewer mental health problems compared to controls	for children; Depressive symptoms: Measured using the 10-item Children’s Depression Inventory Short Form (CDI:S). Self-concept: Measured using the 20-item Tennessee Self-Concept Scale (TSCS)for caregivers. Clinically relevant psychological symptoms: Measured using an adapted 34-item Brief Symptom Inventory (BSI), Parenting stress: Measured using the Parenting Stress Index Short Form (PSI-SF), which assesses parental distress, parent-child dysfunctional interaction, and complex child domains	The sources indicate that parent peers and community health workers facilitated the intervention, and it was effective; however, there is no explicit information on the direct impact on the LMHWs themselves (e.g. confidence, knowledge, burnout, retention).	The ‘Amaka Amasanyufu’ MFG intervention was found to be effective in reducing depressive symptoms and improving self-concept among children with DBDs, while also reducing parental stress and mental health problems among caregivers.	The COVID-19 pandemic caused significant restrictions (school closures, banning of mass gatherings, travel restrictions), which prevented the study from starting baseline assessments and intervention delivery in the four planned treatment schools.
Klein [25]	The training was led by the supervision team, which consisted of clinical psychologists and doctoral students affiliated with academic institutions.	Lay leaders understood the treatment rationale. They adhered to treatment procedures. They believed the intervention components to be helpful and culturally relevant. There was perfect attendance across groups and high levels of participation.	The study used qualitative content analysis of extracted WhatsApp text messages from the supervision group.	Enhanced psychoeducation and skill development. Lay leaders believed the intervention components were helpful and culturally relevant, implying confidence in delivery.		The study acknowledged that clinical supervision is generally ‘difficult to facilitate in countries with limited providers, resources, and internet infrastructure’. However, the study’s findings indicated that WhatsApp supervision overcame these challenges by being technologically feasible.
Ibrahim [31]	Not specified in the provided excerpts.	• â€¢The paper discusses the current state and the potential for task-shifting to close the mental health gap in service delivery. It highlights that the lack of treatment increases the burden of illness in terms of mortality, morbidity, stigma, and discrimination, and that mental illness is a leading cause of disability globally. Economically, mental disorders lead to reduced productivity, unemployment, loss of wages, and resultant poverty.	Does not include any outcome measures because it is a policy and systems brief report	â€¢Not specified in the provided excerpts. The paper proposes utilizing available human resources through task-shifting	The paper states that one in every five people in countries affected by humanitarian crises suffers from some form of mental disorder. It also notes that more than 75% of people with cognitive, neurological, and substance use conditions in LMIC, especially in conflict and post-conflict settings, do not have access to effective mental health services. The impact includes increased burden of illness, disability, and economic strain at the individual and national levels.	Loss of essential human resources in healthcare due to civil war, with most either fleeing the country or becoming victims
Tilahun [29]	The training was part of a program developed by the Federal Ministry of Health (FMOH) of Ethiopia in collaboration with the Open University UK. The facilitators were likely to be health educators or trainers assigned by the Ministry of Health, regional health bureaus, or partnering academic institutions.	High level of interest and motivation among HEWs to learn about child mental health and developmental needs. Improved knowledge, skills, and attitudes toward child mental health care after the HEAT training. Increased confidence and competence in detecting child mental disorders and providing basic care. Use of training materials: Nearly half of the HEWs reported regularly using mental health training materials in their practice. Community engagement: Approximately 35.6% of HEWs organized mental health awareness-raising meetings within four months of completing their training. Recognition of the burden of child mental disorders is widespread and essential. Reduction in stigma and discrimination: HEWs believed their improved knowledge could help tackle stigma within communities. Acceptability of integrating child mental health care into routine community-based primary health care services. Identification of barriers to service provision, including poor knowledge and skills, stigma, demotivation, unrealistic community expectations, lack of referral services, and institutional constraints such as lack of supervision and resources. Identification of facilitators for integration, including positive attitudes, availability of training materials, motivation, willingness to apply training, and perceived fit with HEWsâ€™ roles.	structured survey for quantitative data and in-depth interviews for qualitative data	The training empowered HEWs to understand and address the problem of child mental disorders, which was considered widespread and a significant burden	Community Outcomes: More children identified and supported; early intervention encouraged. Community Engagement: Increased caregiver involvement and community dialogue on child mental health. Stigma Reduction: Shift in community attitudes; reduced fear and improved understanding.	Participants listed a lack of competence as a barrier to service provision.
Nakimuli-Mpungu [12]	The facilitators included health workers trained to deliver the respective interventions – either GSP or GHE – and they conducted the training sessions for the LHWs.		Major depression: MINI (Mini International Neuropsychiatric Interview) Functioning: Locally developed 5-item Function Assessment Tool Post-traumatic stress symptoms: Locally adapted Harvard Trauma Questionnaire Suicide risk: SAD PERSONS Scale Alcohol use: AUDIT (Alcohol Use Disorders Identification Test) Coping skills: Modified Coping Inventory HIV-related stigma: Brief AIDS-Related Stigma ScaleAdherence to ART: Self-report question on missed medication in the past weekViral load: Clinical chart data; categorized as suppressed or notCost-effectiveness: Depression scores mapped to DALYs via the WHO DALY Calculator and ICER	The source describes that lay health workers were trained and delivered the intervention.	The study found that the effects of GSP were most pronounced among men, a group that typically has low participation in health interventions in low-resource settings. This suggests that integrating GSP into existing HIV services can attract and engage men more effectively, potentially leading to broader community health improvements.Participants in the GSP group experienced significant reductions in depression and sustained improvements in functioning over 12 months. As depression and related mental health issues were alleviated, this likely contributed to overall community well-being by reducing the burden of mental illness.The study also notes reductions in suicide risk, post-traumatic stress symptoms, and hazardous alcohol use among GSP participants. These secondary outcomes suggest a broader community-level impact on mental health and social stability, especially in communities facing adverse circumstances like conflict and displacement.By effectively addressing depression, GSP might improve ART adherence and retention in care, which has implications for community health by reducing HIV transmission and improving viral suppression among people living with HIV (PLHIV). Engaging men and reducing stigma can also enhance community support for HIV-affected individuals.The high participation rate and the acceptability of the group support model demonstrate that community-based delivery of mental health interventions is feasible and can be integrated into routine HIV care, thereby broadening access to mental health support in underserved settings.	Since outcome assessors were not masked to the intervention group, there is a risk that detection bias may have influenced the measurement of outcomes such as depression severity and adherence. The reliance on self-reports for ART adherence may have led to overestimation, as no gold standard exists for adherence assessment, and self-reports can be influenced by social desirability bias. Viral load suppression was assessed retrospectively from clinical files, which included only annual measurements, potentially underestimating the rate of ART failure during the assessment period. The use of a conservative threshold (<1000 copies/mL) may misclassify some patients’ virologic status, thereby affecting the accuracy of outcome prediction. Societal costs, including food and other non-healthcare patient expenses, were not captured, which may underestimate the actual economic impact of the intervention. Additionally, the short follow-up period (only one year) limited the extrapolation of long-term cost-effectiveness. Viral load testing was costly (~$400 per person), which limited the ability to conduct more frequent assessments and societal perspective analyses. This posed a challenge for the comprehensive evaluation of the intervention’s impact on viral suppression. The unblinded outcome assessment and reliance on self-reports introduce potential biases and measurement errors, affecting the robustness of the results. The follow-up period of only one year limits the understanding of the long-term sustainability of intervention effects and cost-effectiveness, particularly regarding viral suppression and mental health outcomes.
Murray [32]	The training was developed and used by the authors themselves.	The apprenticeship model enabled lay counselors to deliver mental health interventions effectively, resulting in positive client outcomes. Intensive supervision enhanced fidelity and sustained the quality of intervention delivery over time. Additionally, the model increased local capacity by training supervisors and counselors, thereby supporting sustainable mental health service systems in low-resource settings.	Client mental health symptoms. Client functioning and disability levels. Counselor fidelity to the intervention protocol. Quality of intervention delivery. Counselor skill competency. Supervision effectiveness. Client symptom monitoring tools. Counselor self-report of session activities and practice assignments. Objective supervisor reports of counselor sessions and feedback quality.	The apprenticeship model enhanced lay counselors’ skills, confidence, and ability to deliver interventions with fidelity through hands-on training and ongoing supervision, thereby supporting their professional growth and local capacity building.	The apprenticeship model improved access to quality mental health services in the community by enabling trained local counselors to provide culturally relevant care, which helped reduce the burden of mental health disorders and strengthened sustainable mental health support systems in low-resource settings.	The reported challenges included supervisor and counselor attrition, limited local capacity to handle clinical emergencies, significant time and resource demands, and the need for supervisors and trainers to share a common language for effective communication and supervision.
Bond [30]	Most supervisors were affiliates of the local implementing partner, FXB Rwanda, and had helped develop training and monitoring tools.	Increased preparedness and confidence, improved quality of delivery, Knowledge gain, Fidelity to the intervention, Enhanced competence	Qualitative key informant interviews Fidelity and competence Self-report	increased confidence Skill Development knowledge gain	built trust, improved family conduct,	technological issues, delayed or insufficient compensation
Barnett [31]	Not specified in this excerpt.	enhance non-specialist delivery of interventions	Not specified in this excerpt.	Aims for non-specialists to deliver interventions with high levels of competence and fidelity. Advocates for training that recognizes the expertise non-specialists bring and is tailored to their skills and needs. Explicit impacts, such as confidence, knowledge gain, burnout, or retention, are not detailed.	Contributes to addressing the global mental health burden and the gap in mental health service provision. Non-specialists promote culturally responsive care within their communities.	The COVID-19 pandemic exacerbates widespread mental health needs and disparities. â Substantial worldwide gap between mental health needs and service provision has been recognized for decades. â—¦ High rates of unmet care (72â€- 93% in LMICs) and similar gaps for marginalized populations in HICs. â COVID-19 pandemic impacts (death, social distancing, job losses) have increased mental health needs
Venturo-Conerly [28]	Study Team Members: This included multiple members of the research team who were involved in designing and delivering the training. Notably, the core team from Nairobi consisted of T.O., D.N., C.M., V.M., and C.W., with some team members consistently participating in interviews and training activities. Undergraduate Trainers: The training was led by undergraduate students, most of whom had prior research experience and studied psychology. They received support and supervision from doctoral-level advisors and were trained to facilitate the sessions independently. Doctoral-level advisors provided oversight, support, and guidance during the development of training protocols and supervision strategies. They were available for consultation if participant risks or protocol deviations arose. Study Team Members During Role Plays: During practice sessions, study team members observed and provided immediate feedback as facilitators of the role plays	Improved service delivery: Lay provider satisfaction: effective mental health interventions	Fidelity and Performance Ratings: Assessed through recordings of sessions, rated on six domains: delivering required content, adhering to details, thoroughness, skillfulness, clarity, accessibility, and purity. These ratings used a 7-point scale, with reliability assessed via Gwet’s AC2 statistic, and average ratings were shown to be very good to excellent across domains. Lay Provider Feedback: Quantitative measures included ratings on a scale from 1 to 7 for preparation, usefulness, comfort, clarity, and overall impressions, collected immediately post-training and after intervention completion. Qualitative feedback was gathered on the strengths and weaknesses of training and supervision to capture subjective experiences and perspectives. Performance of Lay Providers in Role-Playing: Assessed using a detailed rubric during role-plays to determine adequacy, with follow-up practice provided for those who struggled, ensuring proper preparedness.Supervision Efficacy: Evaluated through the frequency and content of supervision meetings, with ratings of supervision quality and support.	Many lay providers reported personal growth. Quantitative ratings of the training were overwhelmingly positive, indicating high levels of satisfaction.	Enhanced Accessibility and Reach of Mental Health Support: Training local lay providers enabled the delivery of mental health interventions (Shamiri program) within secondary schools, making support more accessible to adolescents and reducing barriers related to stigma and resource limitations in Kenyan communities. Improvement in Student Well-being and Mental Health: The intervention’s focus on culturally appropriate modules, such as growth mindset, gratitude, and value affirmations, selected for their efficacy and cultural relevance, suggests a potential for a positive impact on students’ mental health, which in turn benefits the broader community.Community Engagement and Capacity Building: Training local community members as lay providers fostered community involvement and capacity building, potentially leading to sustained mental health support beyond the study period, and strengthening community resilience.Positive Perceptions and Acceptance: Feedback from lay providers highlighted feelings of personal growth and confidence, which could translate into more community engagement and trust in mental health initiatives.	Logistical and Timing Issues: Participants expressed concerns about the logistics of training, including transportation and the location of training sessions, which impacted accessibility. Many suggested a more central location to reduce travel burdens, especially in traffic-heavy areas like Nairobi. Training Content and Style: While feedback on training style was generally positive, some participants found certain aspects challenging, such as the need for more frequent protocol reviews and the initial intimidation of role-playing exercises. One provider suggested reviewing the protocol more frequently during supervision to reinforce learning. Time Burden and Preparation: Lay providers noted that they would have benefited from more time to learn and ask questions before leading groups, indicating that the brief 10-hour training might have been intensive for some without sufficient preparatory time.Supervision and Support: Concerns were raised about supervision logistics, including the timing of reimbursements for transportation and the frequency of reminders. Resource Constraints:The challenge of balancing ‘day jobs’ with work as a lay provider, along with limited resources such as transportation funding and logistical support, posed barriers to the consistent and effective delivery of intervention.
Sensoy-Bahar [24]	The facilitators of the training were team members who had already been trained in the intervention by the Principal Investigator (PI), who had developed the original intervention.	Lay providers reported feeling prepared to deliver the intervention after receiving the training. They expressed enjoyment in seeing the families engaged and participating actively during the intervention delivery	semi-structured in-depth interviews	Facilitators were motivated to participate due to the perceived benefits for their community and families.	The lay providers themselves perceived the intervention as benefiting their community and the families within it.	Facilitators had concerns regarding their lack of previous experience in delivering such interventions. They also expressed concerns about the time commitment required for the program.
Wakida [33]	Mental health specialists from Mbarara Regional Referral Hospital.	Empowerment of HC III-level primary health care providers to treat and manage some common mental disorders instead of referring all cases to HC IV, due to their similar cadre of health professionals (except for the absence of a medical doctor). Need to modify HMIS registers to include mental health problems for better recording and monitoring at the primary care level. Requirement for more straightforward guidelines on psychotropic medicines supply, especially at HC III level, to specify which medicines are for refills versus new treatments. Recognition of cost implications for scaling up the intervention nationwide, particularly concerning the training and supervision components at lower-level health centers. Summarized guidelines and register modifications alone are insufficient; training and supervision are crucial to successfully integrating mental health services into primary healthcare.	Feasibility of the educational intervention (measured by PHCPsâ€™ ability and willingness to use the summarized Uganda Clinical Guidelines and modified registers). Acceptability of the intervention by primary health care providers (PHCPs), reflected in their feedback and attitudes toward the tools and training. Uptake and utilization of the Uganda Clinical Guidelines (UCG) for mental health in primary health care settings. Changes in clinical practice, such as increased confidence in assessing, managing, or referring patients with mental health disorders. Adherence to the mental health assessment and management protocols as outlined in the summarized guidelines and checklist. Documentation accuracy of mental health cases in the modified HMIS registers. Perceived workload and time constraints associated with integrating mental health care into routine services. Frequency and quality of training and supervision support provided to PHCPs. Drug stock management issues related to psychotropic medicines supply and usage patterns.	PHCPs attributed their success in using the summarized UCG to the training, suggesting an improved ability or confidence in using the guidelines.	Increased Mental Health Awareness and Access: Health workers observed that more patients were presenting at health facilities with mental health concerns following the intervention. Community members appeared more aware that mental health care was available at the PHC level, improving early identification and access. Early Identification and Management: The training enabled PHC providers to recognize signs and symptoms of common mental disorders earlier. This improved the capacity of HC III and IV facilities to diagnose and manage mental health conditions that previously might have gone unrecognized. Reduced Unnecessary Referrals: By equipping HC III staff to handle mild to moderate mental illnesses, the need for referrals to higher-level facilities decreased. This improved continuity of care and reduced burdens on district hospitals. Improved Patient Outcomes: Providers reported observing better responses to treatment among patients initiated on psychotropic medication at the PHC level. There was a perception that functionality and well-being improved for some patients who accessed care locally. Reduced Stigma through Provider Engagement: Some health workers shared that the intervention challenged their own assumptions and reduced stigma around mental illness, which may positively influence community attitudes.	Shortage of Psychotropic Medications Frequent stock-outs of essential psychotropic drugs limited the ability of providers to treat patients effectively. HC III facilities, in particular, lacked adequate medication supplies. Limited Time and Heavy Workload Healthcare workers reported being overwhelmed by competing clinical responsibilities, leaving them with little time to focus on mental health assessment and care. Inadequate Space and Privacy Lack of private consultation rooms hindered confidential discussions with patients experiencing mental health issues. Lack of Confidence in Managing Severe Cases. Despite training, some providers still felt unprepared or hesitant to manage more complex mental health presentations, especially in the absence of doctors at the HC III level. Incomplete Integration into Health Management Information System (HMIS) The HMIS registers did not include mental health conditions, making it difficult to track and report mental health data systematically. Stigma and Misconceptions. Some health workers still hold stigma about mental illness, which could affect the quality of care and community engagement. Lack of Role Clarity for Mental Health within PHC Health workers were unsure about the scope of their responsibility in mental health care, particularly regarding medication refills and follow-up care. Need for Ongoing Supervision and Support There was a concern that without continued supervision, providers might revert to previous practices or feel unsupported in handling mental health cases.
Karungi [34]		Before the training, LHWs had limited knowledge about dementia-related support, with activities primarily focused on general support, such as nutrition and health education. After the training, LHWs gained a basic understanding of dementia and began actively sensitizing their communities. LHWs reported feeling more comfortable working with people with dementia. A notable change in the attitude of family members towards people with dementia was reported. â—¦ The conclusion suggests that with enhanced capacity, LHWs may be able to support community-based management for people with dementia	Outcomes were assessed through a qualitative evaluation of LHWs’ knowledge on community-based management and care for people with dementia, as well as an assessment of their experiences during the eight-week implementation period.	After the training, LHWs gained more knowledge about dementia-related support, performing only general support, nutrition, and health education, and felt more comfortable working with people with dementia	LHWs began sensitizing communities about dementia. A reported outcome was a notable change in the attitude of family members towards people with dementia	LHWs reported difficulties in differentiating the signs of early dementia from superstitious beliefs
Asher [36]						
Mutamba 2013	This information is not provided in the source.	Included both symptom reduction (depression, PTSD), child development, psychosocial functioning, and some adverse events, with mixed but generally positive indications of intervention effectiveness despite methodological limitations.	These measures collectively captured symptom severity, functional status, developmental progress, and psychosocial factors to evaluate the effectiveness of interventions led by lay health workers.	LMHWs demonstrated potential as a valuable workforce to extend mental health services in resource-limited settings, but their success depended on adequate training, supervision, and support systems.	There was evidence of the effectiveness of interventions, specifically a reduction in symptoms of depression and improved child mental development.	Most studies (*n* = 13) involved small sample sizes Many studies had methodological issues, including unclear allocation concealment, lack of blinding, and incomplete handling of outcome data, which affected the strength of the evidence. The diverse intervention types, roles of LHWs, and outcome measures made it challenging to compare studies or perform meta-analyses.

Academic and clinical institutions were frequently engaged as key facilitating actors. For example, Nakimuli-Mpungu et al. [22] reported training led by specialists from Makerere University in collaboration with Uganda’s Ministry of Health, using a training-of-trainers model, which supported capacity building and scalability of the intervention. Similarly, HIV care providers from The AIDS Support Organization [TASO] facilitated group HIV education sessions, while Nakimuli-Mpungu et al. [12] noted that health workers trained in group support psychotherapy [GSP] or HIV education subsequently trained LMHWs.

Parents, teachers, and trainers were also involved as facilitators. Brathwaite et al. [23] reported training of parent peers and community health workers by team members under the principal investigator’s guidance. Klein et al. [27] described training and supervision provided by clinical psychologists and doctoral students, supplemented by WhatsApp for ongoing supervision. Venturo-Conerly et al. [37] used a layered model where undergraduate trainers worked under doctoral-level advisors, with role plays and feedback provided by research staff. Sensoy Bahar et al. [38] also described research team members, initially mentored by the principal investigator, as training facilitators.

Governmental and health system led models were also common. Tilahun et al. [29] reported training of health extension workers through Ethiopia’s Health Extension Program, developed in collaboration with the Open University UK. Wakida et al. [33] noted training led by specialists at Mbarara Regional Referral Hospital in Uganda, while Bond et al. [30] reported supervision conducted by FXB Rwanda, which also developed training and monitoring tools.

Supervision models varied considerably across the board, reflecting resource availability and contextual differences. Murray et al. [32] depicted an apprenticeship supervision model stressing intensive, hands-on support to enhance fidelity and foster sustainable local capacity. Klein et al. [27] illustrated the utility of digital platforms such as WhatsApp for real-time supervision and peer support in low resource contexts. Venturo-Conerly et al. [37] incorporated structured fidelity monitoring, role-play evaluations, and ongoing supervision meetings, which were rated highly by LMHWs. Wall et al. [26] further reinforced the indispensable role of supervision in sustaining worker motivation and mitigating burnout, although challenges related to role conflict and workload stressors persisted.

### Outcomes

The scoping review revealed a broad range of reported outcomes, spotlighting the effectiveness, feasibility, and sustainability of LMHW delivered interventions across East African contexts.

### Client level outcomes

Some studies have documented significant improvements in mental health symptoms and functioning. Group support psychotherapy [GSP] delivered by LMHWs reduced rates of major depression, improved functional status, and yielded secondary benefits such as increased self-esteem, reduced post-traumatic stress, lower stigma, and improved adherence to antiretroviral therapy and viral suppression among people living with HIV in Uganda, right [22]. Multi-family group interventions for children resulted in reduced depressive symptoms and improved self-concept, while caregivers reported decreased stress and fewer mental health concerns [23]. Other trials demonstrated gains in quality of life and psychosocial functioning, including an 81% increase in physical quality of life, a 21% increase in mental quality of life, and a 97% reduction in internalized stigma [21].

### Provider level outcomes

In six studies, lay providers consistently reported improvements in knowledge, confidence, competence, and satisfaction following training and supervision. Across studies, these gains were more pronounced in programs that combined structured, multi-component training with ongoing supervision and practical learning methods, such as role-plays, supervised practice, and competency assessments [30,37,38]. Increased fidelity to intervention delivery, preparedness, and engagement in group and family sessions were also reported in interventions that incorporated regular supervision and feedback mechanisms. Providers further noted that culturally adapted interventions enhanced acceptability and perceived relevance, contributing to greater adherence to treatment protocols [27].

In addition, health extension workers [HEWs] in Ethiopia demonstrated improved capacity to detect and manage child mental health conditions, which was associated with targeted training and supportive supervision structures embedded within the health system [29]. Similarly, training programs that included condition-specific modules, such as dementia care, were linked to increased provider confidence and effectiveness in supporting affected families [34]. Overall, the evidence suggests that integrated training and supervision models-particularly those emphasizing experiential learning and contextual adaptation-were associated with stronger provider outcomes across studies.

### Service delivery and system level outcomes

Numerous interventions have reported increased service delivery and care retention. Some studies noted zero loss to follow-up over 12 months and increased HIV-related knowledge and social support [20,21,]. Apprenticeship supervision models reinforced fidelity and built a sustainable local capacity [32]. Systemic outcomes included improved capacity of primary health centers to manage common mental health conditions and recognition of the need for stronger health information systems, medication supply chains, and sustainable financing [33].

### Motivation

Lay providers reported high motivation and intrinsic satisfaction in their roles, often linking their participation to professional growth, personal fulfillment, and a sense of community contribution [26].

### Acceptability

Across studies, interventions were generally reported to be acceptable to both providers and communities, particularly when they were culturally adapted and aligned with local norms and community expectations [31,35].

### Feasibility

The reviewed interventions were also described as feasible within community health systems, particularly where they were integrated into existing structures and supported by local health teams and simplified delivery models [31,35].

Impacts of Training and Supervision on Lay Mental Health Workers (LMHWs) benefited both professionally and personally from their participation. Professionally, they have reported significant increases in competence, confidence, and cultural sensitivity across a range of interventions, including GSP, child mental health care, and dementia support [22,30,32,34]. HEWs have gained skills in child mental health detection and management and demonstrated increased confidence in using training materials and guidelines [29,33]. Community health and support workers amplified their professional identity and social recognition [21]. Personally, LMHWs reported improved self-esteem, intrinsic motivation, and coping skills, deriving satisfaction from witnessing positive outcomes within their communities [20,37,38].

### Community level outcomes

Community-wide benefits were evident across multiple studies. Interventions increased engagement with HIV and mental health services, improving testing, care initiation, adherence, and retention [20–]. In addition, mental health – specific outcomes included reductions in depressive symptoms, anxiety, post-traumatic stress, and psychological distress, as well as improvements in psychosocial functioning and wellbeing in several studies. GSP and other culturally tailored approaches were associated with reductions in depressive symptoms, hazardous alcohol use, suicide risk, and posttraumatic stress, while enhancing functional capacity and resilience. Child and family focused interventions reduced caregiver stress, increased mental health awareness, and supported early identification of at-risk children [23,29]. Stigma reduction was a recurring theme, with LMHWs and health workers facilitating sensitization and improving community attitudes toward mental health [29,33,34]. Finally, interventions stimulated community empowerment by strengthening social support networks, engaging families and men in health promoting behaviors, and enhancing the visibility and legitimacy of community-based mental health services [21,26,31,32].

### Challenges

The scoping review identified multiple challenges affecting the delivery, uptake, and sustainability of LMHWs delivered mental health interventions in East Africa.

### Program reach and engagement

Several studies have reported limited program reach and engagement due to logistical barriers, competing responsibilities, and insufficient involvement of community health workers [20,37,38]. Attrition among supervisors, counselors, and LMHWs was also noted, alongside difficulties sustaining participant engagement [32]. In some pilot trials, the absence of control groups restricted the ability to attribute outcomes directly to the interventions [21].

### Training and competence

Challenges related to training quality and adequacy were frequently observed. Some LMHWs reported that training sessions were too brief, lacked sufficient protocol review, or did not provide adequate opportunities to practice role-playing exercises [37,38]. Limited prior experience among lay facilitators and low confidence in managing complex cases, particularly in contexts with minimal specialist support, further constrained intervention quality [29,33,34].

### Resource and infrastructure constraints

Resource limitations were often cited, including inadequate transportation support, a lack of private consultation spaces, and insufficient availability of psychotropic medications, especially at lower-level health centers [33]. Weak integration of mental health into health management information systems [HMIS] hindered the monitoring of outcomes [33]. Broader systemic issues, including civil unrest and shortages of healthcare personnel, further affected service delivery in certain settings [35].

### Methodological and evaluation limitations

Studies have repeatedly reported methodological constraints that limited the generalizability of findings. For instance, small sample sizes, lack of blinding, reliance on self-reported measures, and short follow-up durations [22,]. Some studies also identified measurement challenges, such as accurately assessing antiretroviral therapy adherence and viral suppression, while failing to capture broader economic or societal outcomes [21,]. The COVID-19 pandemic further disrupted intervention delivery and data collection, particularly for school-based programs [23,31].

### Psychosocial and occupational challenges

Lay providers reported high workloads, emotional stress, and burnout associated with delivering mental health services [26,30]. Role conflict, unclear responsibilities, and experiences of stigma further undermined confidence and effectiveness in some cases [33,34].

### Financial and sustainability concerns

Sustainability emerged as a key concern. While stipends were reported to enhance short-term motivation and retention, questions remained about long-term financial viability [21]. Challenges included delays or inadequate compensation, reliance on external funding, and the resource-intensive nature of some supervision models, which limited scalability [27,29].

## Discussion

This scoping review examined how lay mental health workers [LMHWs] are trained and supervised to deliver community-based mental health interventions across five East African countries, synthesizing reported outcomes, facilitators, and implementation challenges. For consistency, this review uses the term lay mental health workers (LMHWs) to refer to all non-specialist providers delivering mental health interventions across the included studies. The literature mostly demonstrates significant promise: LMHWs delivered programs were frequently associated with improvements in client symptoms and functioning, enhanced provider knowledge and confidence, and system-level gains in service delivery. At the same time, the evidence base was heterogeneous in both scope and quality. Supervision practices were inconsistently described and rarely evaluated as independent implementation components, while critical questions about long-term sustainability, cost, and workforce wellbeing remain unresolved.

Training approaches varied considerably, ranging from brief, skills-focused workshops [e.g. two-day, 8–10-hour formats] to intensive multi-module programs, diplomas, and apprenticeship models [22,25,29,37]. Programs that integrated didactic instruction with experiential learning strategies, such as role-plays, supervised pilot sessions, competency assessments, and refresher training tended to report stronger provider outcomes, plus increased competence, fidelity, and confidence [22–24]. However, the heterogeneity in training duration, content, and pedagogy complicates the determination of a minimum competency threshold or an evidence-based ‘dose’ of training required to ensure safe and effective deliveries across diverse contexts. We were not surprised that workshop-type formats were the most used for training lay leaders in our targeted settings. Implementation of science literature suggests that workshops are often preferred because they allow for interactive group learning, faster dissemination, and efficient use of limited resources, such as time, money, and personnel to run the training [39]. For example, in Project HEAL, lay peer community health advisors trained through in-person workshops showed comparable outcomes to those trained via web-based methods in implementing health education workshops [39]. In addition, hybrid strategies combining workshops with feedback or coaching have been found to be effective in trials such as Project MIMIC, which compared different didactic workshop modalities alongside supervision and role-play fidelity assessments.

An important consideration emerging from this review is the heterogeneity in the definition and roles of lay mental health workers (LMHWs). Across studies, LMHWs included unpaid community volunteers, peer supporters, community health workers, and salaried paraprofessionals embedded within health systems. This variation has important implications for training and supervision design.

For example, unpaid volunteers may require supervision approaches that prioritize motivation, retention, emotional support, and fidelity to intervention protocols. In contrast, salaried paraprofessional cadres may benefit more from supervision that emphasizes clinical skill development, accountability, and integration into formal service delivery systems. The lack of differentiation between these groups in many studies limits the ability to identify context-specific supervision models and suggests the need for more tailored implementation strategies in future work.

Supervision strategies comprised of hierarchical clinical oversight, peer- and team-based models, apprenticeship and coaching approaches, and digitally supported modalities [e.g. WhatsApp groups] [24,27,32,33]. Hybrid approaches combining professional oversight with peer support and group reflection have emerged as realistic models that balance fidelity with feasibility in resource limited and geographically dispersed settings. Despite these innovations, supervision was rarely evaluated as a standalone implementation strategy. Most studies described supervisory frequency and modality but did not assess how supervision type, intensity, or fidelity directly influenced provider performance or client outcomes.

However, this variability in supervision approaches also reflects an important strength, namely the flexibility to adapt models to diverse resource constraints, geographical settings, and workforce capacities. In several contexts, flexible supervision arrangements such as peer support, hybrid models, and digital platforms enabled continued service delivery in settings where formal clinical supervision was limited or unavailable.

Various studies have reported meaningful client-level improvements, such as reductions in depressive symptoms, improved antiretroviral therapy [ART] adherence, and reduced stigma, as well as system-level gains consisting of improved retention in care [21]. The close integration of HIV and mental health services in several studies reflects the strong bidirectional relationship between HIV and psychological distress in East African contexts. Mental health challenges such as depression, trauma, and stigma were frequently reported among people living with HIV, and in turn were associated with poorer adherence, retention, and treatment outcomes. LMHW-delivered interventions therefore operated at this intersection by addressing both psychological wellbeing and HIV care engagement, demonstrating the value of integrated, task-shared models in high HIV burden settings. Providers also noted an enhanced professional identity, motivation, and skill acquisition [26,30]. Nonetheless, many studies were constrained by methodological limitations, including small sample sizes, short follow-up periods, reliance on self-report, and lack of control groups or blinding. These methodological limitations restricted the ability to draw causal inferences from the included studies and limited the generalizability of their findings across East African and similar low-resource settings [22,]. Importantly, studies that incorporated fidelity monitoring or competency assessments tended to report more positive outcomes, calling attention to the value of structured training combined with ongoing quality assurance.

Academic institutions, health system stakeholders, and research teams often played key roles in initiating and supporting training and supervision activities, frequently employing training-of-trainers and apprenticeship models to build local capacity [22,23,29]. Digital platforms, such as WhatsApp, were highlighted as feasible tools for real-time supervision and peer support [24,25].

The findings from this review should also be understood within broader Global South task-sharing approaches, where similar LMHW models have been implemented in South Asia and other parts of Africa. For example, community-based psychosocial interventions in countries like as India and Pakistan have demonstrated the effectiveness of structured task-sharing models combined with ongoing supervision and strong integration into primary care systems. Similarly, programs in Southern Africa have highlighted the importance of community embeddedness, where trust, cultural alignment, and social proximity of lay providers enhance engagement and service uptake. Across these contexts, contextual factors, such as poverty, health system fragmentation, workforce shortages, stigma, and reliance on external funding, consistently shape implementation outcomes. These similarities suggest that the successes and challenges observed in East Africa are not isolated but reflect broader structural conditions influencing LMHW programs across the Global South.

However, persistent barriers related to resource limitations [e.g. transportation, private spaces, and medication access], weak integration with health management information systems [HMIS], inconsistent or delayed remuneration, role conflict, and burnout threatened scalability and sustainability [21,30,33]. Moreover, community participation in the design and delivery of supervision remains limited, despite the community-based standards of most interventions.

Although task-sharing approaches are widely described as cost-effective, the findings of this review highlight that supervision systems require substantial and sustained investments. Effective supervision is not cost-free, as it depends on trained supervisors, protected time within clinical workloads, transportation resources, and sometimes digital infrastructure for remote support.

In many included studies, supervision responsibilities were added onto existing clinical duties without dedicated funding or workload adjustment, raising concerns about long-term sustainability. Without explicit budgeting for supervision time and infrastructure, there is a risk that supervision quality may decline over time, potentially undermining intervention fidelity and workforce retention. Strengthening the economic infrastructure for supervision is therefore essential for scaling and sustaining lay mental health worker programs in resource-constrained settings.

## Implication

This scoping review highlights lay mental health workers (LMHWs) as a critical and scalable workforce for addressing mental health service gaps in low-resource East African settings, particularly where specialist shortages are severe. Across the included studies, LMHW-led interventions were associated with improved service access, reduced stigma, and enhanced client outcomes such as symptom reduction, treatment adherence, and psychosocial functioning (e.g.[20]). At the workforce level, LMHWs reported gains in knowledge, confidence, and professional identity, suggesting that task-sharing approaches can generate benefits for both service users and providers.

A key implication is that training and supervision are not one-off activities but must be designed as continuous, structured, and context-responsive processes embedded within health systems. Evidence from included studies suggests that programs combining didactic teaching with experiential methods (e.g. role-plays, supervised practice, and competency assessments) were more likely to report improved provider competence and fidelity (e.g. [22,23]). This supports implementation science evidence that active, skill-based learning approaches are more effective than purely lecture-based models.

Importantly, the review also highlights the value of co-design approaches, where LMHWs, service users, and community stakeholders are actively involved in shaping training content, supervision structures, and delivery strategies. Although rarely reported in the included studies, participatory design methods are increasingly recognized in global mental health as improving cultural relevance, acceptability, and sustainability of interventions. In the East African context, co-design may be particularly important given variations in language, literacy levels, and community norms, and could strengthen ownership and intervention fitness.

Supervision emerges as a non-negotiable component for maintaining intervention quality and LMHW wellbeing. However, the evidence suggests that supervision is often inconsistently implemented and weakly evaluated, despite its importance for maintaining fidelity, addressing challenges, and preventing burnout. Hybrid models combining in-person, group-based, peer, and digital supervision (e.g. WhatsApp-based support) appear promising in improving feasibility in resource-constrained settings (e.g. [24,27,32]). Nevertheless, stronger evidence is needed to determine how supervision intensity and structure influence outcomes over time.

Beyond training and supervision, LMHW-led programs demonstrate broader system and community-level benefits, including improved service uptake, increased mental health awareness, and reduced stigma (e.g. [12–22]). These outcomes are likely facilitated by LMHWs’ embeddedness within communities, which enhances trust and engagement. However, contextual factors-including resource constraints, weak health system integration, role ambiguity, and inconsistent remuneration-continue to limit scalability and sustainability.

Finally, the review underscores the need for stronger evaluation designs to establish the long-term effectiveness and cost-effectiveness of LMHW-led interventions. Future research should also address sociocultural and structural determinants of implementation success, including stigma among health professionals, system readiness, and the integration of LMHWs into formal health structures. Importantly, more participatory and co-designed research approaches are needed to ensure that training and supervision models are not only evidence-based but also locally owned and contextually grounded.

## Limitation

This scoping review, while providing a comprehensive mapping of the training and supervision of lay mental health workers (LMHWs) in East Africa, has several limitations inherent in both the included literature and the scoping review methodology.

At the same time, a key strength of this review is its systematic synthesis of a relatively fragmented and under-consolidated evidence based on LMHW training and supervision in East Africa. By bringing together studies across multiple countries and intervention types, the review offers a regionally specific overview that had not previously been synthesized at this level of detail. Additionally, the inclusion of diverse study designs (qualitative, quantitative, and mixed-methods) allowed for a more comprehensive understanding of both implementation processes and reported outcomes, which is particularly valuable in an emerging and context-dependent field.

First, the methodological quality and reporting of the included studies varied significantly. Across the literature, common limitations included small sample sizes, lack of blinding, reliance on self-reported outcomes, and short follow-up periods, all of which constrain the generalizability of findings (e.g. [22,]). In addition, several studies provided limited implementation details, particularly regarding training duration, supervision frequency, and facilitator roles (e.g. [31,35,36]). While this limits comparability and replication, it also reflects the early-stage and implementation-focused nature of many LMHW programs, where documentation of process details is often secondary to service delivery. Importantly, in-depth qualitative descriptions of LMHW experiences in some studies do provide rich contextual insight, which can be a strength for understanding how interventions operate in real-world settings, even if not always standardized.

Second, the review is constrained by its geographic focus on East Africa. While this allows for contextual specificity and policy relevance, it may limit transferability to other low- and middle-income settings with different health system structures, cultural norms, and workforce configurations. Several included studies also reported contextual disruptions such as COVID-19-related service interruptions and civil unrest, which affected implementation and data collection (e.g. [23,31,35]).

Finally, a key limitation emerging from the evidence is the limited examination of long-term outcomes. Few studies have assessed sustainability, LMHW retention, or financial viability beyond initial implementation or donor funding cycles (e.g. [21,27,30]). There is also limited documentation of community involvement in supervision design, despite the community-embedded nature of many interventions. This restricts our understanding of how participatory governance mechanisms may influence long-term program success.

### Key takeaways for training and supervision of lay mental health Workers

Across the included studies, several recurring implementation patterns emerged that may inform future program design and policy development for lay mental health worker (LMHW) interventions in East Africa.

**Training parameters**:

- Training duration ranged from short workshops (2–5 days) to multi-week or modular programs, suggesting that both brief and extended models can be effective when combined with experiential learning.
- Programs that included role-plays, supervised practice, and competency assessments demonstrated stronger provider confidence and fidelity.
- Training embedded within existing health systems (e.g. HIV programs or primary care structures) enhanced scalability and sustainability.

**Supervision parameters**:

- Effective supervision was typically ongoing rather than one-off, with weekly to monthly contact being most common in higher-functioning models.
- Hybrid supervision approaches (in-person + peer + digital tools such as WhatsApp) have improved feasibility in low-resource and geographically dispersed settings.
- Structured supervision using checklists, fidelity monitoring tools, and feedback loops was associated with stronger implementation quality.

**Implementation insight**:

- Supervision was most effective when integrated into existing health system workflows and supported by trained clinical supervisors with protected time.

## Conclusion

This scoping review synthesizes the current evidence on the training and supervision of lay mental health workers [LMHWs] delivering community-based interventions in East Africa. The findings reveal a diverse and innovative landscape where a variety of actors, including academic institutions, research teams, government health systems, and NGOs, are successfully training and supporting LMHWs to bridge the significant mental health treatment gap.

This review demonstrates that training programs, though heterogeneous in duration and method, are effective in equipping LMHWs with the necessary knowledge, skills, and confidence to deliver mental health interventions. From intensive multi-day workshops to shorter, skill-based training, these programs are consistently associated with improved provider competence and self-efficacy. Similarly, supervision strategies, ranging from hierarchical clinical oversight to peer-based and technology-supported hybrid models, are crucial for maintaining intervention fidelity, providing psychosocial support, and preventing burnout among LMHWs. The promising use of digital platforms like WhatsApp highlights a viable path for sustainable supervision in resource-limited and geographically dispersed settings.

The reported outcomes highlight the profound impact of these programs across multiple levels. At the client level, interventions led by LMHWs result in significant improvements in mental health symptoms, functional status, and adherence to treatment for conditions like HIV. At the provider level, LMHWs experience professional growth, increased cultural sensitivity, and personal fulfillment. Crucially, at the community level, these initiatives lead to increased mental health service uptake, reduced stigma, enhanced early identification of problems, and stronger social support networks, fostering greater community empowerment and resilience.

However, this promise is tempered by significant challenges, including logistical barriers, resource constraints, methodological limitations in the evidence, and concerns over the long-term sustainability of funding and support for LMHWs. Moving forward, the field must prioritize the development of more standardized and rigorously evaluated training and supervision models. Future efforts should focus on integrating mental health into primary care systems more vigorously, actively involving communities in program design, and implementing sustainable financing and supervision strategies. By addressing these challenges, the immense potential of LMHWs can be fully realized, making a substantial contribution to achieving just and accessible mental health care for all in East Africa and similar low-resource contexts.

## Supplementary Material

PRISMA Updated.docx

## Data Availability

No new data were created or analyzed in this study. Data supporting the findings of this review are available within the cited literatureThe completed PRISMA-ScR checklist for this review is available as a supplementary file.
